# The use of genomic information increases the accuracy of breeding value predictions for sea louse (*Caligus rogercresseyi*) resistance in Atlantic salmon (*Salmo salar*)

**DOI:** 10.1186/s12711-017-0291-8

**Published:** 2017-01-31

**Authors:** Katharina Correa, Rama Bangera, René Figueroa, Jean P. Lhorente, José M. Yáñez

**Affiliations:** 1 Facultad de Ciencias Veterinarias y Pecuarias, Universidad de Chile, Av Santa Rosa 11735, La Pintana, Chile; 2 Aquainnovo, Cardonal s/n Lote B, Puerto Montt, Chile

## Abstract

Sea lice infestations caused by *Caligus rogercresseyi* are a main concern to the salmon farming industry due to associated economic losses. Resistance to this parasite was shown to have low to moderate genetic variation and its genetic architecture was suggested to be polygenic. The aim of this study was to compare accuracies of breeding value predictions obtained with pedigree-based best linear unbiased prediction (P-BLUP) methodology against different genomic prediction approaches: genomic BLUP (G-BLUP), Bayesian Lasso, and Bayes C. To achieve this, 2404 individuals from 118 families were measured for *C. rogercresseyi* count after a challenge and genotyped using 37 K single nucleotide polymorphisms. Accuracies were assessed using fivefold cross-validation and SNP densities of 0.5, 1, 5, 10, 25 and 37 K. Accuracy of genomic predictions increased with increasing SNP density and was higher than pedigree-based BLUP predictions by up to 22%. Both Bayesian and G-BLUP methods can predict breeding values with higher accuracies than pedigree-based BLUP, however, G-BLUP may be the preferred method because of reduced computation time and ease of implementation. A relatively low marker density (i.e. 10 K) is sufficient for maximal increase in accuracy when using G-BLUP or Bayesian methods for genomic prediction of *C. rogercresseyi* resistance in Atlantic salmon.

## Background

Sea lice are common marine external parasites that belong to the order of Copepoda. *Caligus rogercresseyi* is the major sea lice species of interest in the southern hemisphere, while *Lepeophtheirus salmonis* is the major species of interest in the northern hemisphere. Both species affect Atlantic salmon (*Salmo salar*) and rainbow trout (*Oncorhynchus mykiss*) [1]. *C. rogercresseyi* parasitize farmed salmonids and wild fish in Chile and constitute one of the main threats for salmon aquaculture in one of the major salmon producing countries [2, 3].

Sea lice can cause skin lesions, osmotic imbalance, and increased susceptibility to bacterial and viral infections through suppression of host immune responses and damage to the host skin [4, 5]. Sea lice may also play a role in the transmission of different fish pathogens [1, 6]. Large economic losses occur as a result of reduced feed conversion and growth, indirect mortality, loss of product value, and treatment costs [1]. The worldwide cost of the control of this parasite in the salmon farming industry was estimated in 2009 to be US$480M [7].

Different chemicals are used to control sea lice, but increasing resistance to antiparasitic drugs has been reported [8]. An alternative method to control sea lice is selective breeding, which has been proposed as a feasible option to improve disease resistance in several livestock and aquaculture species [9–13]. To be selected efficiently, a trait must exhibit significant genetic variation. Genetic parameters have been estimated for *C. rogercresseyi* resistance in Atlantic salmon. Lhorente et al. [14] estimated a heritability (*h* ^2^) of 0.32 for sessile *C. rogercresseyi* count on fins in Atlantic salmon, while Yáñez et al. [15] and Correa et al. [16] estimated a *h* ^2^ of 0.12 for the same trait using pedigree and molecular information, respectively. These low to moderate heritabilities indicate that it is feasible to include these traits in the breeding program for Atlantic salmon.

Using a 50 K genotyping array, a recent study conducted by Correa et al. [16] provided evidence for a polygenic architecture of resistance to *C. rogercresseyi*. Similarly, Houston et al. [17, 18] used a 200 K single nucleotide polymorphism (SNP) genotyping array to assess the genetic architecture of resistance to *L. salmonis* and observed polygenic inheritance for this trait also. The polygenic nature of these traits suggests that major quantitative trait loci (QTL) contributing to the genetic variance are unlikely and thus, that marker-assisted selection (MAS) may not be the most appropriate way to include molecular information for selection [19]. In such cases, it is possible to use a dense SNP genotyping panel to obtain genomic estimated breeding values (GEBV) of animals that lack own phenotypic records, an approach known as genomic selection (GS) [20]. The difference between MAS and GS is that MAS uses only single nucleotide polymorphisms (SNPs) that are significant in an association analysis, whereas GS uses all SNPs without having to set a significance threshold [19]. Genetic gain can be increased in salmon breeding programs through the use of molecular markers to calculate GEBV, which are more accurate than EBV calculated using pedigree-based methods [21–23].

Several GS methodologies to predict GEBV have been proposed, which differ in prior assumptions on the distribution of the effects of the SNPs [19]. For example, in genomic best linear unbiased prediction (G-BLUP), marker effects are assumed to be normally distributed, which implies that there is a large number of QTL underlying the trait, each with a small effect [20]. In Bayesian Lasso, marker effects are assumed to follow a double exponential distribution [24], which implies that a large proportion of the markers have an effect close to zero and a small proportion have moderate to large effects [19]. In Bayes C, only a fraction of the markers is assumed to have an effect and, all these are assumed to have a common variance, instead of locus-specific variances [25].

Genomic selection has been tested with real data in salmonid species in a few studies [26–29] that have evaluated growth, fillet color and disease resistance traits. In this study, we compared accuracies of pedigree-based BLUP EBV with those obtained with Bayesian and G-BLUP methods for *C. rogercresseyi* resistance, using data from 2404 individuals and a 50 K genotyping array.

## Methods

### Phenotypic records

Phenotype data were available for 2404 Atlantic salmon smolts from 118 families (i.e. progeny of 118 dams and 40 sires) from the breeding population of Salmones Chaicas, Xth Region, Chile. These individuals were experimentally challenged with *C. rogercresseyi*. The average number of fish per family was 22 and ranged from 9 to 24, and the average weight of fish was 274.9 g (SD = 90.6 g). The fish were tagged with passive integrated transponders, acclimated, and then distributed to three replicate tanks. The challenge test was carried out as described previously [14–16]. Briefly, infestation with the parasite was carried out by using 13 to 24 copepodids per fish and stopping the water flow for 6 h after infestation. The challenge lasted 6 days; then, fish were euthanized and fins from each fish were collected and fixed for processing and lice counting. The resistance trait was defined as the count of sessile lice per fish on all fins after the infestation period, since that is highly representative of the total lice count on the fish [14]. Experimental tank and final body weight were recorded for each fish.

### Genotypes

Genotype data were available from a previous study [16]. Briefly, genomic DNA was extracted from fin clips using a commercial kit (DNeasy Blood and Tissue, Qiagen), quality controlled and quantified. All phenotyped fish were genotyped using a 50 K SNP Affymetrix^®^ Axiom^®^ myDesign™ Genotyping Array designed by Aquainnovo and the University of Chile. More details about the SNPs included in the 50 K array are in Correa et al. [30] and Yáñez et al. [31, 32].

The genotypes were quality controlled using the Affymetrix Genotyping Console and the SNPolisher R package following the Axiom^®^ Genotyping Solution Data Analysis Guide [33]. Additional quality control steps were conducted by filtering out SNPs with a Hardy–Weinberg equilibrium test *p* value less than 0.00001, an SNP call rate lower than 0.95 and a minor allele frequency lower than 0.01.

### Estimation of breeding values

The conventional pedigree-based approach, P-BLUP, was used as the control for genomic evaluations, and EBV for each individual were obtained using a linear mixed model implemented in BLUPF90 [34]. The model used was as follows:

$${\mathbf{y}} = {\mathbf{X}}{\boldsymbol{\upbeta}} + {\mathbf{Tg}} + {\mathbf{e}},$$

where **y** is a vector of phenotypes (lice count on fins), **β** is a vector of fixed effects (mean, tank and body weight effects), **g** is a vector of random additive polygenic genetic effects that follows a normal distribution ~$N({\mathbf{0, A}}\sigma_{{\mathbf{g}}}^{2} )$, **X** and **T** are incidence matrices, **A** is the pedigree additive relationship matrix, and **e** is the residual [35].

The genomic EBV (GEBV) for each individual were estimated using three different GS models: G-BLUP, Bayesian Lasso and Bayes C. G-BLUP was as implemented in the BLUPF90 software [34]. G-BLUP is a modification of the BLUP method, where the numerator relationship matrix **A** is replaced by a genomic relationship matrix **G**, as described by VanRaden [36].

For Bayesian methods, marker and additive polygenic effects were estimated jointly using the following model implemented in the GS3 software [37]:

$${\mathbf{y}} = {\mathbf{X}}{\boldsymbol{\upbeta }} + {\mathbf{Z}}{\boldsymbol{\upalpha }} + {\mathbf{Tg}} + {\mathbf{e}},$$

where **α** is a vector of random marker allele substitution effects and **Z** is the corresponding incidence matrix. The Gibbs sampler was run using 100,000 iterations with a burn-in of 20,000 iterations. Priors were drawn from double—exponential and scaled—inverse *χ* ^2^ distributions, for Bayesian Lasso and Bayes C, respectively. Bayesian GEBV were estimated as the sum of the polygenic and marker effects.

Pedigree (P-BLUP) and genomic (G-BLUP) heritabilities were calculated using the AIREMLF90 software [34] as follows: $h^{2} = \sigma_{{\mathbf{g}}}^{2} /(\sigma_{{\mathbf{g}}}^{2} + \sigma_{{\mathbf{e}}}^{2} )$, where $\sigma_{{\mathbf{g}}}^{2}$ is the estimate of the additive genetic variance and $\sigma_{{\mathbf{e}}}^{2}$ is the estimate of the residual variance.

### Comparison of models

The different models were compared using a fivefold cross validation scheme. Briefly, all genotyped and phenotyped animals were randomly separated into five validations sets, which were predicted one at a time by masking their phenotypes and using the remaining animals as training set to estimate the marker effects. Thus, for each validation run, the dataset was split into a training set (80%) and a validation set (20%). To reduce the stochastic effects, this cross-validation analysis was replicated 10 times. Accuracy was used to assess the performance of each model and was estimated as:

$$\varvec{R}_{{\varvec{EBV},\varvec{BV}}} = \varvec{ }\frac{{\varvec{R}_{{\varvec{EBV},\varvec{ y}}} }}{\varvec{h}},$$

where $\varvec{R}_{{\varvec{EBV},\varvec{ y}}}$ is the correlation between the EBV of a given model (predicted for the validation set using information from the training set) and the actual phenotype, while ***h*** is the square root of the pedigree-based estimate of heritability [26, 38]. To test prediction accuracies obtained by using various SNP densities, 0.5, 1, 5, 10 and 25 K SNPs were selected from the full set as follows. First, 500 SNPs that had a level of linkage disequilibrium (LD, measured as r^2^) less than 0.2 and were homogeneously distributed across the genome were selected among the SNPs that passed quality control. Then, SNPs with a homogeneous distribution across the genome were added to the first 500 SNPs to create the 1 K panel. The same procedure was reiterated to create the 5, 10 and 25 K panels. Accuracies were then calculated for each model and SNP density, compared to those obtained with the P-BLUP model, and the relative increase in accuracy was assessed.

## Results and discussion

A total of 36,616 SNPs passed the genotyping quality control. An average lice count on fins of 5.1 (SD = 4.4) and an average final body weight of 281 g (SD = 92.8) were obtained. The average lice number per family ranged from 2.3 to 11.3. The pedigree-based estimate of heritability of lice count on fins was equal to 0.10, which is consistent with a previous study on the same population [15], while the marker-based estimate of heritability using the 37 K full set was equal to 0.11.

**Fig. 1:**
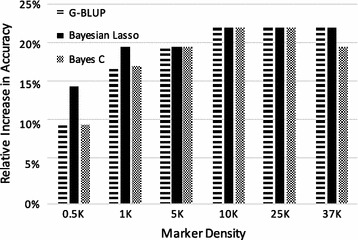
Relative increase in EBV prediction accuracy of genomic selection models for *Caligus rogercresseyi* resistance compared to a pedigree-based BLUP model

In all cases, prediction accuracies obtained with the GS models were higher than those obtained with the pedigree-based model, which had an accuracy of 0.41 (Table 1). It is interesting to note that as few as 500 SNPs were sufficient to increase the accuracy of breeding value predictions. Further tests using even lower marker densities may be interesting to assess at which marker density accuracy of genomic prediction drops below that based on pedigree information. The relative increases in accuracy of the different methods for each SNP density are shown on Fig. 1. Comparing the results for all SNP densities shows that the different GS models behaved similarly, with accuracies ranging from 0.45 to 0.5. In general, accuracies increased moderately with increasing SNP density and achieved asymptotic values when 10 K or more SNPs were used. Bayesian Lasso performed slightly better than the other methods at lower SNP densities, but at higher densities, prediction accuracy was similar for all three GS methods. We observed a slight decrease in accuracy with Bayes C when using the full SNP dataset, which was not observed with G-BLUP or Bayesian Lasso. G-BLUP mostly captures genetic relationships between animals, whereas Bayes C uses the LD between SNPs and QTL to calculate predictions. In Bayes C, only a fraction of the SNPs is assumed to have an effect on the trait, and thus when adding more SNPs for calculating predictions, the use of redundant information (SNPs in high LD with each other) [39] might lead to incorrect selection of the subset of SNPs with an effect from the whole set. The accuracies obtained in our study on Atlantic salmon were not as high as those reported for other livestock species, which can be even higher than 0.85 in some cases. Nevertheless, accuracies were similar to those determined in previous studies on Atlantic salmon for *L. salmonis* resistance, for example, Tsai et al. [29] reported accuracies ranging from 0.4 to 0.6. To further increase the accuracy of GEBV predictions in Atlantic salmon breeding programs, it may be necessary to increase the number of individuals and generations in the training population [40].

**Table 1:** Accuracies for sea lice (*Caligus rogercresseyi*) resistance obtained using different models and different marker densities

Marker density	P-BLUP	G-BLUP	Bayesian Lasso	Bayes C
0	0.41	–	–	–
500	–	0.45	0.47	0.45
1000	–	0.48	0.49	0.48
10,000	–	0.50	0.50	0.50
25,000	–	0.50	0.50	0.50
50,000	–	0.50	0.50	0.49

P-BLUP accuracy only applies to 0 markers as it does not use marker information

We found that the three GS methods had similar GEBV prediction accuracies, in spite of the different priors used in each model. This is the first study that aims at evaluating the performance of GS methods for *C. rogercresseyi* resistance in Atlantic salmon. Moreover, it is the first study that evaluates Bayesian method performance for sea lice resistance using a high-density SNP array and different SNP densities in this species. We demonstrate that it is possible to increase the accuracy of breeding value predictions for *C. rogercresseyi* resistance using SNP information.

Prediction accuracies obtained by using genome-wide marker information were higher than those obtained by using only pedigree information to account for the relationship between individuals. These results are in line with other studies that evaluated the performance of GS for different traits in Atlantic salmon [26, 27]. For example, using only pedigree information, Ødegård et al. [26] estimated an EBV reliability (defined as $R_{EBV, y}^{2} /h^{2}$) of 0.34 and 0.36, which increased by up to 50 and 20% for *L. salmonis* resistance and fillet color when using 22 K SNPs in an admixed population. For growth traits, Tsai et al. [27] achieved an increase in accuracy by up to 20% when using 5 K SNPs. Similarly, Tsai et al. [29] achieved an increase in accuracy of 27% when using 5 K SNPs for *L. salmonis* resistance. A recent study in rainbow trout [28] reported similar prediction accuracies between P-BLUP and GS methods using a 57 K genotyping array for bacterial cold water disease resistance.

Our results show that SNP density had a moderate impact on prediction accuracy for all GS methods tested in this study, and that as few as 500 SNPs were sufficient to increase EBV accuracy over P-BLUP. Moreover, 10 K may suffice to obtain maximum increases in accuracy for this trait and this population.

This study used real data from a breeding population and the results suggest that GS may be effective in improving resistance to this sea lice species. Further studies aimed at evaluating the use of low-density panels and imputation strategies are necessary to determine the minimum required SNP density for GS and thus reduce genotyping costs.

## Conclusions

It is possible to improve prediction accuracy for *C. rogercresseyi* resistance in Atlantic salmon by using different densities of SNPs selected from a 37 K panel. By applying different genomic prediction approaches, we showed that as few as 500 SNPs were sufficient to increase the accuracy of EBV to a higher value than that obtained from pedigree-based methods. We found that the maximum increase in accuracy was obtained with 10 K SNPs when using G-BLUP and Bayesian methods.
